# De Novo Design and Development of a Nutrient-Rich Perfusate for Ex Vivo Lung Perfusion with Cell Culture Models

**DOI:** 10.3390/ijms241713117

**Published:** 2023-08-23

**Authors:** Lei Huang, Ravi N. Vellanki, Zhiyuan Zhu, Bradly G. Wouters, Shaf Keshavjee, Mingyao Liu

**Affiliations:** 1 Latner Thoracic Surgery Research Laboratories, Toronto General Hospital Research Institute, University Health Network, Toronto, ON M5G 1L7, Canada; huanglei917@outlook.com (L.H.); zhuzhiyuan19920531@126.com (Z.Z.); shaf.keshavjee@uhn.ca (S.K.); 2 Princess Margaret Cancer Centre, Campbell Family Institute for Cancer Research, University Health Network, Toronto, ON M5G 1L7, Canada; ravi.vellanki@uhnresearch.ca (R.N.V.); brad.wouters@uhnresearch.ca (B.G.W.); 3 Departments of Surgery, Institute of Medical Science, Temerty Faculty of Medicine, University of Toronto, Toronto, ON M5G 1A8, Canada

**Keywords:** modification of perfusate, ex vivo organ perfusion, cell culture, translational research

## Abstract

Ex vivo lung perfusion (EVLP) has increased donor lung utilization through assessment of “marginal” lungs prior to transplantation. To develop it as a donor lung reconditioning platform, prolonged EVLP is necessary, and new perfusates are required to provide sufficient nutritional support. Human pulmonary microvascular endothelial cells and epithelial cells were used to test different formulas for basic cellular function. A selected formula was further tested on an EVLP cell culture model, and cell confluence, apoptosis, and GSH and HSP70 levels were measured. When a cell culture medium (DMEM) was mixed with a current EVLP perfusate—Steen solution, DMEM enhanced cell confluence and migration and reduced apoptosis in a dose-dependent manner. A new EVLP perfusate was designed and tested based on DMEM. The final formula contains 5 g/L Dextran-40 and 7% albumin and is named as D05D7A solution. It inhibited cold static storage and warm reperfusion-induced cell apoptosis, improved cell confluence, and enhanced GSH and HSP70 levels in human lung cells compared to Steen solution. DMEM-based nutrient-rich EVLP perfusate could be a promising formula to prolong EVLP and support donor lung repair, reconditioning and further improve donor lung quality and quantity for transplantation with better clinical outcome.

## 1. Introduction

Lung transplantation has become the effective treatment for patients with end-stage lung disease, and the number of lung transplant procedures increased year by year [1]. However, donor lungs are more vulnerable than other solid organs, and the utilization rate is less than 20%; thus, donor lung shortage has remained as a critical challenge that has led to high mortality of patients on waiting lists [2]. Ex vivo lung perfusion (EVLP) is a new technology for donor lung assessment before transplantation. Marginal donor lungs were ventilated and perfused outside of the body, and its clinical application has greatly increased the utilization of donor lungs [3,4]. EVLP has been further developed as a platform for donor lung repair, such as the use of thrombolysis [5], high-dose antibiotics [6], lung surfactant [7], anti-inflammation drugs [8], anti-viral treatments [9,10], and reconditioning [11]. Moreover, EVLP has been tested for delivery of therapeutic gene [12], mesenchymal stromal cells [13], and regulatory T cells for immunomodulation [14].

However, currently, clinical EVLP is limited for 6 h [3], and most experimental EVLP with pig lungs are about 12 h [8,15]. To prolong EVLP, a cross-circulation strategy has been developed, of which an ex vivo donor pig lung is supported by a host pig for up to 36 h [16]. Using this model, acid-aspiration-induced donor lung injury was partially recovered with injury-specific treatment within the 36 h time frame [17]. This technique was further extended to 4 days [18]. Under immunosuppression and recombinant cobra venom factor to limit innate and adaptive immune response, Hazain et al. have used this model to support human lungs declined from clinical programs for 24 h [19]. These breakthroughs paved a path towards xenogeneic cross-circulation for potential donor lung repair; however, the clinical implementation of the cross-circulation technique is challenged by many technical and ethical issues.

The EVLP system is a technique whereby a donor lung is placed in a protective dome and ventilated and perfused with a special perfusate, at normothermic temperatures [3]. Steen solution is the most-used perfusate for EVLP. It contains buffered electrolytes, glucose, Dextran-40, and albumin (Table 1). A recent metabolomic study demonstrated that the lack of nutrients in EVLP perfusate is one of the limiting factors of current EVLP protocol [20]. Slow injection of total parenteral nutrition into the EVLP circuit has extended EVLP to about 24 h [21,22]. Recently, Huang et al. demonstrated that Steen solution alone does not support survival of human lung epithelial and endothelial cells in culture; adding L-alanyl-L-glutamine into Steen solution significantly improved human lung cell functions and extended porcine lung EVLP to longer than 30 h [23]. This work not only indicates that the modification of Steen solution may extend EVLP and enhance clinical EVLP performance, but also demonstrated that cell culture models could be used to screen well-designed formulas as a next generation of EVLP perfusates.

In the three recent studies [21,22,23], nutrients were added to current Steen solution. Instead of modifying Steen solution, we would like to design a new solution with enriched nutrients. We hypothesized that amino acids, vitamins, and other nutritional and cytoprotective components seen in cell culture media can be used to develop new perfusates to support EVLP extensively for donor organ repair and reconditioning.

## 2. Results

### 2.1. Adding DMEM to Steen Solution Rescued Human Lung Cellular Function

**Figure 1:**
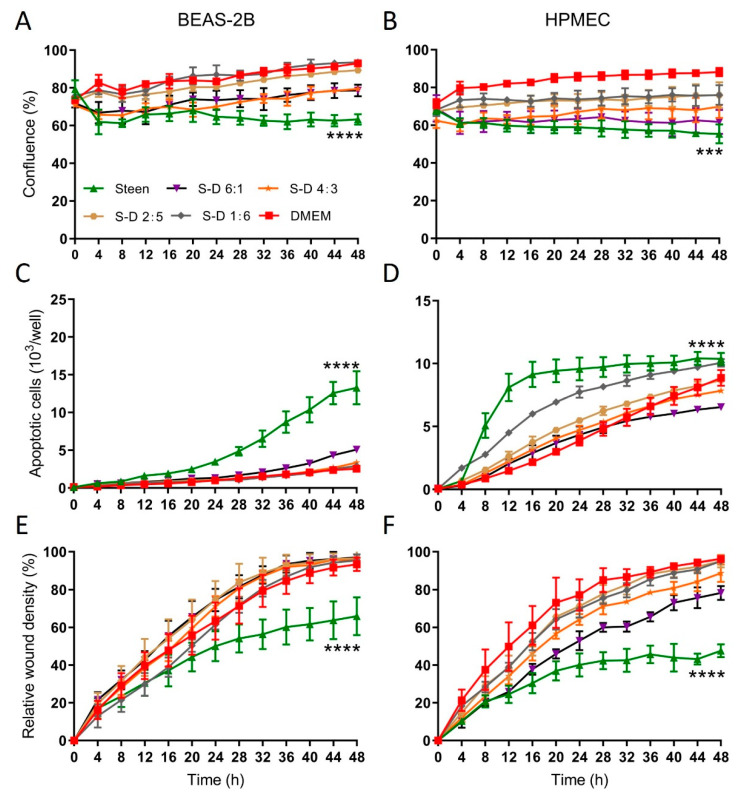
Mixing DMEM with Steen solution in different ratios improved human lung epithelial and endothelial cell functions. Human lung epithelial BEAS-2B cells and human pulmonary microvascular endothelial cells (HPMEC) were cultured in 96-well plates with DMEM plus 10% FBS until sub-confluence, and then switched to Steen solution or DMEM, or both Steen solution and DMEM mixed in different ratios (1:6, 2:5, 4:3, 6:1). Cells were monitored with the IncuCyte^®^ System. (**A**,**B**): Cell confluence; (**C**,**D**): Cell apoptosis; (**E**,**F**): Cell migration; *n* = 4 wells for confluence and apoptosis; *n* = 8 wells for migration studies. All experiments have been repeated at least 3 times. ***: *p* < 0.001 compared with all other groups except S-D 6:1 group; ****: *p* < 0.0001 compared with all other groups.

Our previous study demonstrated that when human lung cells were exposed to Steen solution, cell confluence decreased, apoptosis increased gradually, and cell migration ability was inhibited [23]. Dulbecco’s Modified Eagle Medium (DMEM) is a widely used basal medium for many different mammalian cells [24]. When we added essential amino acids and/or non-essential amino acids from DMEM into Steen solution, the significant drop of pH indicated that the buffering capacity in Steen solution is limited. To test if amino acids and vitamins in a cell culture medium such as DMEM can improve the cellular function of Steen solution, we mixed DMEM with Steen solution in different ratios (6:1, 5:2, 3:4, 1:6). When human lung cells were switched from the regular culture condition (DMEM + 10% FBS) to the Steen solution, the confluence was reduced and maintained at a lower level, while mixing Steen solution with DMEM increased confluence in a DMEM-dose dependent manner (Figure 1A,B). Exposure of human lung cells to Steen solution induced apoptosis, which was significantly reduced when Steen solution was mixed with DMEM in all ratios in both epithelial and endothelial cells (Figure 1C,D). Similarly, cells in Steen solution showed lower migration capacity. Mixing Steen solution with DMEM improved migration of all ratios in both cell types (Figure 1E,F).

### 2.2. Effects of Dextran-40 and Albumin in DMEM on Basic Cellular Function

DMEM is a widely used cell culture medium that contains physiological ions, buffer system, and more abundant amino acids and vitamins (Table 1). We decided to use it as a basic formula to develop new EVLP perfusates. Steen solution contains 5 g/L Dextran-40 and 70 g/L (7%) albumin (Table 1), the former is critical for maintaining osmotic pressure to protect donor lungs from edema during EVLP, while the latter is the most abundant protein of human blood plasma to regulate the oncotic pressure, and it binds to various ligands and carry them around. When Steen solution was mixed with DMEM, Dextran-40 and albumin concentrations were diluted. We added different concentrations of Dextran-40 and albumin to DMEM to test their effects on basic cellular functions.

**Figure 2:**
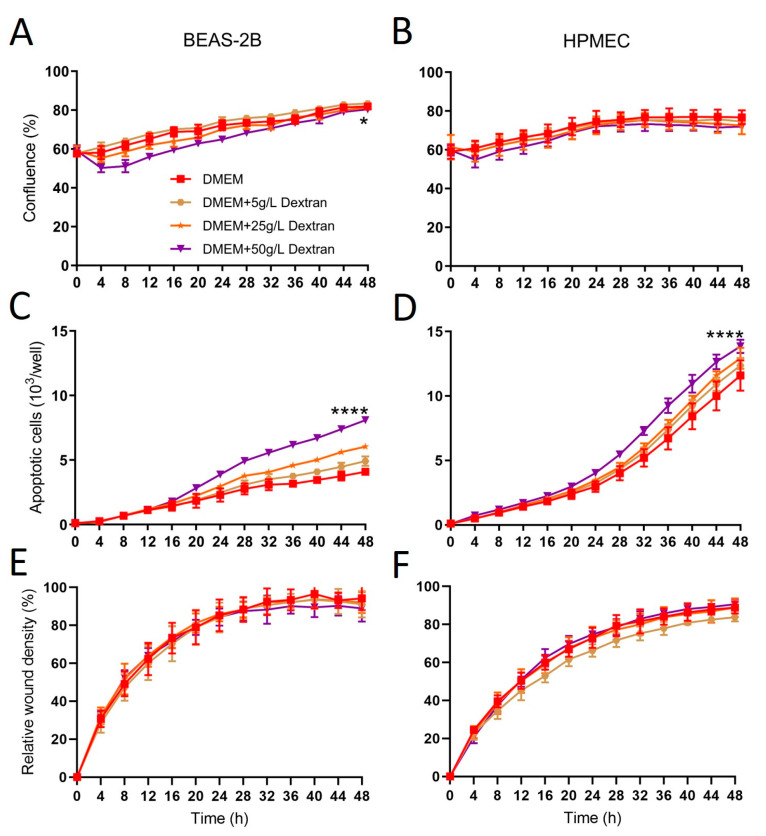
The high dose (50 g/L) of Dextran-40 added to DMEM induced apoptosis in human lung epithelial and endothelial cells. See Figure 1 legend for experimental conditions. Different concentrations of Dextran-40 were added to DMEM (5 g/L, 25 g/L or 50 g/L). (**A**,**B**): Cell confluence; (**C**,**D**): Apoptosis; (**E**,**F**): Cell migration. *: *p* < 0.05; ****: *p* < 0.0001 compared with all other groups.

**Figure 3:**
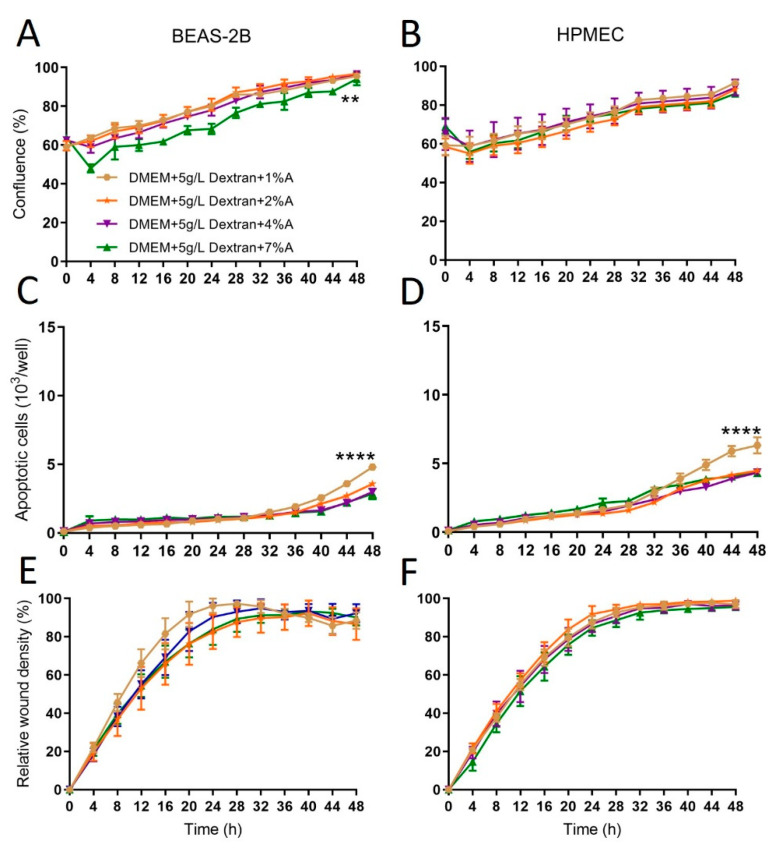
Albumin protected human lung epithelial and endothelial cells from apoptosis. See Figure 1 legend for experimental conditions. Albumin was added to DMEM plus 5 g/L of Dextran-40 solution at different concentrations (1/2/4/7%A refer to albumin concentration from 1–7%). (**A**,**B**): Cell confluence; (**C**,**D**): Apoptosis; (**E**,**F**): Cell migration. **: *p* < 0.01; ****: *p* < 0.0001 compared with all other groups.

DMEM first was added without or with Dextran-40 at 5, 25, 50 g/L. At the higher concentrations (50 g/L), cell confluence was lower than in other groups in epithelial cells (Figure 2A) and showed no difference in endothelial cells (Figure 2B). Compared with DMEM alone, adding Dextran-40 increased apoptosis in a dose-dependent manner in both cell types (Figure 2C,D). Cell migration was less influenced by Dextran-40 concentration (Figure 2E,F). Since higher concentration of Dextran-40 showed certain negative impacts on cellular functions, we kept Dextran-40 concentration at 5 g/L, the same as used in Steen solution and then tested the effects of albumin at different concentrations (1, 2, 4, 7%). The confluence of epithelial cells was significantly lower with 7% of albumin compared with other groups, and no significance was noted in endothelial cells (Figure 3A,B). Interestingly, apoptosis was very low in all groups, except slightly higher apoptosis was observed at the end of the 48 h study period in 1% of the albumin groups in both cell types (Figure 3C,D), suggesting albumin provides certain protection to cells. Cell migration was not significantly affected by albumin concentrations (Figure 3E,F).

Taken together, we decided to use DMEM as a base formula, as it contains multiple essential and non-essential amino acids and a panel well-defined vitamins (Table 1), and it has been used as a cell culture medium to maintain basic cellular function. We also chose to add 5 g/L Dextran-40 and 7% albumin in DMEM, as these concentrations have been proven to be effective in both clinical practice and experimental EVLP studies. We named this new formula as D05D7A solution.

### 2.3. D05D7A Solution Significantly Improved Basic Cellular Function Compared to Steen Solution

**Figure 4:**
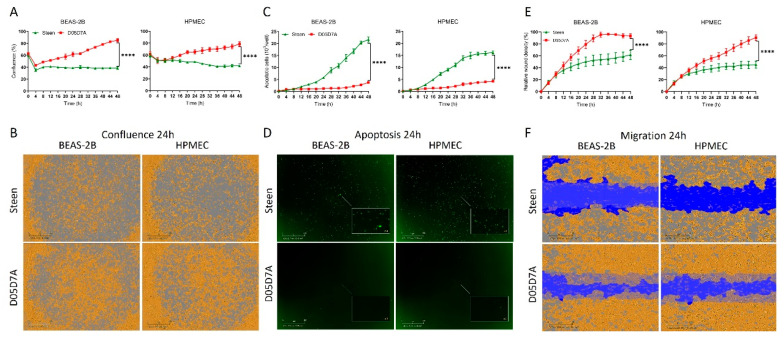
D05D7A solution increased confluence, reduced apoptosis, and enhanced migration of human lung epithelial and endothelial cells compared to Steen solution. See Figure 1 legend for experimental conditions. (**A**,**B**): Cell confluence; (**C**,**D**): Apoptosis; (**E**,**F**): Cell migration. ****: *p* < 0.0001.

We first compared D05D7A solution with Steen solution on basic cellular function on BEAS-2B and HPMEC cells. The cell confluence in the D05D7A solution was increased gradually but remained at lower levels in the Steen solution (Figure 4A). Images of cell confluence at 24 h showed lower confluence for both cell types (Figure 4B). On the other hand, the numbers of apoptosis cells were increased gradually in the Steen solution groups, while they remained under 5% in the D05D7A solution (Figure 4C), which were significantly lower than that in the Steen solution (Figure 4D). Within the first 8 h, cell migration was similar between the two groups; however, after then, the D05D7A groups showed significantly better migration (Figure 4E), and the wound closure was far better than that in the Steen solution groups (Figure 4F).

### 2.4. D05D7A Solution Extended Basic Cellular Function in an EVLP Cell Culture Model

**Figure 5:**
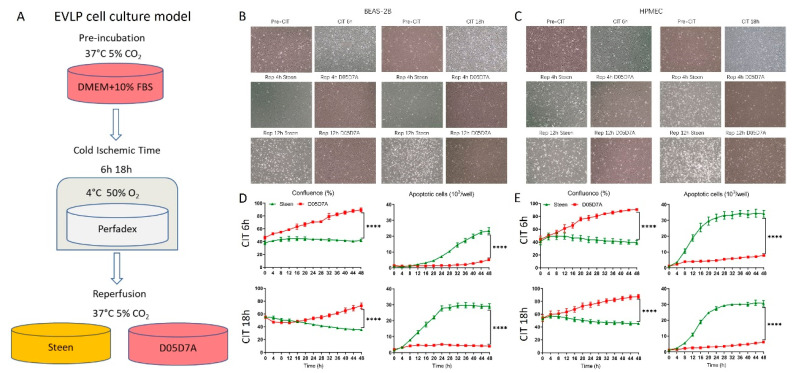
D05D7A solution increased confluence and reduced apoptosis in human lung epithelial and endothelial cells compared to Steen solution in an ex vivo lung perfusion (EVLP) cell culture model. (**A**) EVLP cell culture model (see details in the main text). (**B**,**C**): BEAS-2B and HPMEC cells were cultured in 6-well plates in DMEM plus 10% FBS until sub-confluent. Cells were imaged (100×) before being exposed to cold ischemic time (Pre-CIT), after 6 h cold ischemic time (CIT 6 h), or 18 h (CIT 18 h), and after reperfusion for 4 h (Rep 4 h) or 12 h (Rep 12) in Steen or D05D7A solution. (**D**,**E**): BEAS-2B and HPMEC cells were cultured in 96-well plates (*n* = 6 wells/condition) and continuously monitored for 48 h with the IncuCyte^®^ System. All experiments have been repeated at least 3 times. ****: *p* < 0.0001.

An EVLP cell culture model was recently developed [23]. Cells were cultured in regular DMEM + 10% FBS at 37 °C with 5% CO_2_ until sub-confluent, and then switched to cold preservation solution at 4 °C with 50% O_2_ to simulate cold static storage of donor lung, and then switched to either Steen solution or D05D7A solution at 37 °C to simulate EVLP (Figure 5A). The 6 h cold ischemia time (CIT) represents current clinical practice for donor lung preservation, while 18 h CIT is a model of ischemia-reperfusion injury.

In both lung cell types, the images taken at CIT 6 h, or at CIT 18 h, and after simulated EVLP at 4 h were comparable between the Steen and D05D7A groups, with few floating cells (Figure 5B,C). However, after 12 h of simulated EVLP, much more floating cells can be seen in the Steen groups in both cell types, especially in cells after CIT 18 h, in comparison with those in the D05D7A groups (Figure 5B,C). The cell confluence and apoptosis were quantified with the IncuCyte^®^ System. In epithelial cells, after CIT 6 h, confluence was recovered immediately after cells were switched back to the D05D7A solution, while remained as no improvement in the Steen solution. After CIT 18 h, recovery of confluence in the D05D7A group was delayed until 20 h but the confluence continued to decrease in the Steen group (Figure 5D). Similar results are also found in endothelial cells (Figure 5E). More strikingly, during simulated EVLP, apoptosis was induced in both the epithelial and endothelial cells after either CIT 6 h or CIT 18 h in the Steen groups, while the apoptotic rates remained at very low levels in the D05D7A groups for a continuous 48 h (Figure 5D,E).

**Figure 6:**
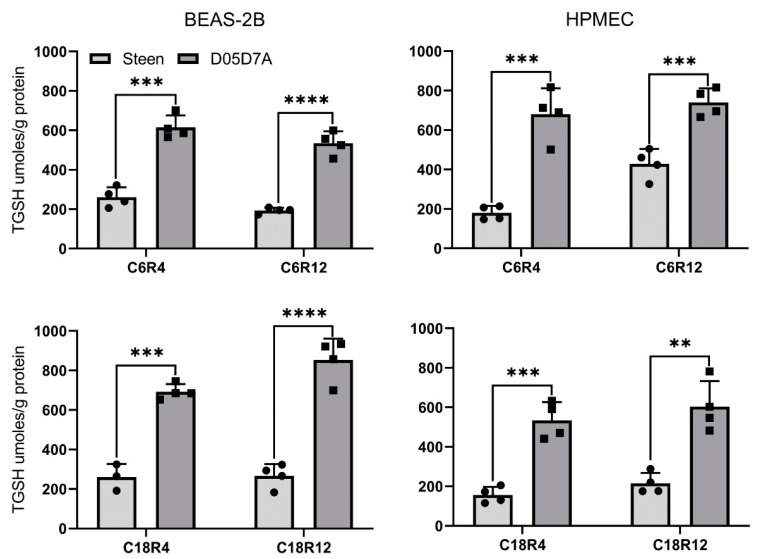
D05D7A solution enhanced total GSH production compared to Steen solution during simulated EVLP in cell culture. See Figure 5 legend for experimental conditions. C6R4: CIT 6 h and reperfusion 4 h, C6R12: CIT 6 h and reperfusion 12 h, C18R4: CIT 18 h and reperfusion 4 h, C18R12: CIT 18 h and reperfusion 12 h. Statistics: Unpaired t test, n = 4 wells/group, **: *p* < 0.01, ***: *p* < 0.001, ****: *p* < 0.0001 between two groups.

The very low apoptosis in the D05D7A groups indicates cytoprotective effects. Thus, we further explored the underlying mechanisms. Glutathione is the most abundant and important intracellular antioxidant. It plays a key role in detoxification of toxins, removal of oxygen-reactive species, and regulation of the redox state of cells, and helps to prevent and reverse cellular damage [24]. The total GSH production at CIT 6 h and CIT 18 h followed by simulated EVLP for 4 h or 12 h was significantly higher in the D05D7A solution groups compared to that in the Steen solution groups in both BEAS-2B and HPMEC cells (Figure 6).

**Figure 7:**
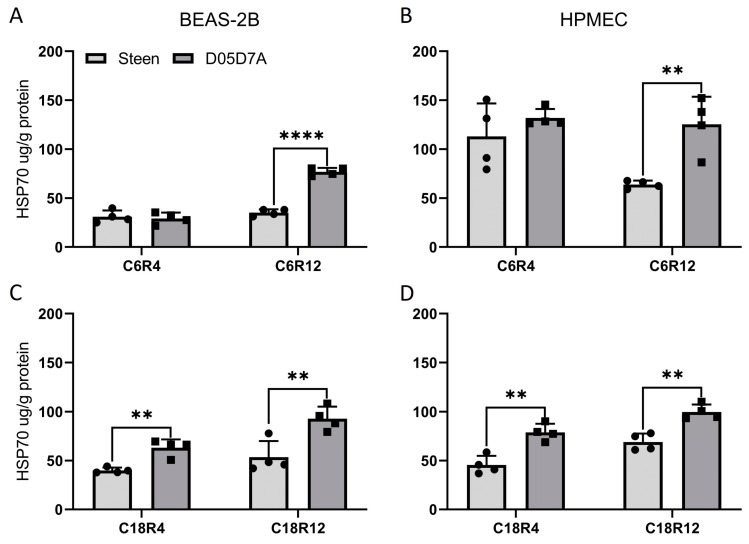
D05D7A solution enhanced HSP70 production compared to Steen solution during simulated EVLP in cell culture. See Figure 5 legend for experimental conditions. C6R4: CIT 6 h and reperfusion 4 h, C6R12: CIT 6 h and reperfusion 12 h, C18R4: CIT 18 h and reperfusion 4 h, C18R12: CIT 18 h and reperfusion 12 h. (**A**,**B**): HSP70 protein level after CIT 6 h followed by simulated EVLP for 4 h and 12 h in BEAS-2B and HPMEC cells; (**C**,**D**): HSP70 protein level after CIT 18 h followed by simulated EVLP for 4 h and 12 h in BEAS-2B and HPMEC cells; Statistics: Unpaired *t* test, *n* = 4 wells/group, **: *p* < 0.01, ****: *p* < 0.0001 between two groups.

Heat shock protein 70 (HSP70) is crucial for de novo folding of nascent polypeptides and interaction with key regulators of many signal transduction pathways controlling cell homeostasis, proliferation, differentiation, and cell death. It inhibits apoptosis acting on both the caspase-dependent and caspase-independent pathways [25]. In the D05D7A-treated cells, the HSP70 protein level after CIT 6 h followed by simulated EVLP for 12 h was significantly higher than that of the Steen solution-treated cells in both cell types (Figure 7A,B). After CIT 18 h followed by either EVLP 4 h or 12 h, the HSP70 protein levels were significantly higher in both cell types treated with D05D7A than that with Steen solution (Figure 7C,D).

## 3. Discussion

Using regular cell culture, we designed and developed a new formula for EVLP perfusate, which is based on DMEM, Dextran-40, and albumin. We then used an EVLP cell culture model and tested the cytoprotective effects of the formula and its potential mechanisms.

### 3.1. De Novo Design EVLP Perfusate

A unique feature of the present study is that we designed a new EVLP perfusate instead of modifying the currently used Steen solution. Buchko et al. continuously infused TPN (amino acids, lipids, and vitamins) to EVLP perfusate mixed with red blood cells [22]. Similarly, Takahashi et al. used a syringe pump to inject TPN (amino acids, vitamin concentrate, and trace elements) to EVLP circuit [21]. Therefore, TPN was added as extra nutrients to EVLP perfusate, not as a modified Steen solution. Huang et al. added L-alanyl-L-glutamine into Steen solution, which significantly improved cellular function and extended porcine lung EVLP to more than 30 h [23]. However, compared with the nutrients added by the Buchko and Takahashi groups, many different nutrients (amino acids, lipids, vitamins, trace elements) could be added to the EVLP perfusate. How to select them and mix them with different combinations is a difficult decision.

To speed up the design and development of a new EVLP perfusate, as a proof-of-principle study, DMEM (a cell culture medium) was chosen as a base formula, and additional Dextran-40 and albumin were added. It contains multiple amino acids (including L-alanyl-L-glutamine), vitamins, and other components (Table 1). This formula protected human lung epithelial and endothelial cells from cold ischemia/warm reperfusion-induced apoptosis, with increased total GSH (antioxidant) and HSP70 chaperon protein (with anti-apoptosis function). This strategy overcomes the limited buffering capacity of Steen solution. It demonstrates the possible de novo design and development of a new perfusate, instead of modifying Steen solutions.

The components of Steen solution are similar to a widely used lung preservation solution, Perfadex^®^ (Table 1), which contains 50 g/L of Dextran-40. During the development of Steen solution, when 50 g/L of Dextran-40 was tested, lung injury was noted (see Steen solution patent). Interestingly, we also observed that when 50 g/L of Dextran-40 was added to DMEM, it also reduced the cell confluence (Figure 2A). Moreover, the use of Dextran-40 is associated with a dose-dependent increase in apoptosis of both epithelial and endothelial cells (Figure 2C,D). Therefore, high doses of Dextran-40 should be avoided, especially when used together with albumin, as both reagents can influence oncotic pressure. The normal range of albumin in the blood is from 34 g/L to 54 g/L. The 70 g/L of albumin in the Steen solution is to maintain a hyperosmotic condition. Interestingly, we found that the apoptosis rate was higher when a low concentration of albumin (1%) was used (Figure 3C,D), suggesting that higher concentrations of albumin may have cytoprotective effects.

We have compared formulas among DMEM, M199, and RPMI-1640 media in a recent publication [26]. Each of these media contain different types and concentrations of amino acids, vitamins, and other components. These media were developed with different purposes and applications. Learning from these formulas may help to design and develop EVLP perfusates. Designing clinically used new EVLP perfusate requires systematic literature review, rational design, high throughput testing and selection, and final pre-clinical and clinical trials.

In “cross-circulation” studies, the ex vivo organ was supported by a host animal [16,17,18,19]. To maintain organ survival for a prolonged time and to have repairing capacity, blood components (red blood cells, plasma, serum, selected growth factors, and cytokines) may have to be considered. Therapeutic drugs should be given when necessary.

### 3.2. Cell Culture Models Enables High-Throughput Screening for New Formulas

EVLP experimentation is extremely expensive, which has limited the related research. Cell culture models play crucial roles in lung-related research, as they are cost effective and easy to use, enabling relatively high throughput screening studies. Several cellular and molecular mechanisms for ischemia–reperfusion injury (such as necroptosis) [27,28] and effective therapeutic drugs (such as alpha 1 antitrypsin, PKCδ inhibitors) [27,29] discovered from cell culture models have been further confirmed with animal lung transplant models [30,31,32]. These models have also been used to develop new lung preservation solutions [26]. Recently, cell culture models have been used to test the biological effects of EVLP perfusate, Steen solution [23,33]. The EVLP cell culture model we developed [23] adds the cold ischemia and warm reperfusion to cells, thus, allowing for the testing of responses of cells to related challenges. Activation of pathways related to inflammatory responses and cell death have been found in human lung tissue after reperfusion, as well as in EVLP [34]. Similar results have been found in human lung epithelial and endothelial cells in culture [35]. A CIT-time dependent increase in cell death (floating cells and apoptosis) was observed in the present study, which was at least partially inhibited by D05D7A solution, which could be attributed, at least partially, to the higher levels of GSH and HSP70 in the cells.

Even though D05D7A solution showed protective effects on both human lung epithelial and endothelial cells, these two cell types have different responses to Steen solution, simulated cold preservation, and warm EVLP. This helps us to understand the cellular responses in a cell-type specific manner. The two cell types have distinct transcriptomic gene signatures, and their responses to simulated cold ischemia and warm reperfusion are also very different at gene expression levels [35]. The cell culture models give opportunities to determine these differences, as each cell type may play different roles in ischemia–reperfusion injury, as well as in the EVLP process.

The current EVLP cell culture model can be further developed to mimic physiological conditions seen in the lung. For example, cyclic mechanical stretch and shear stress should be considered to simulate ventilation and perfusion of the lung. New technologies such as “lung-on-a-chip” [36,37] may be used to develop “EVLP-on-a-chip”. Lung tissue slide culture could be used to mimic complex three-dimensional structures of the lung tissue with multiple cell types.

### 3.3. Limitation of the Present Study and Future Direction

The major limitation of the present study is that we only performed cell culture studies and the final formula was not validated with the porcine lung EVLP model. However, we have previously demonstrated that the addition of L-alanyl-L-glutamine alone extended EVLP significantly [23,33], and the current D05D7A solution contains multiple amino acids including L-alanyl-L-glutamine. We expect its performance would be better. With DMEM as an example, integrant of other formulas would be considered through further studies. Only the best candidates should be further tested with animal models to save the cost and the use of animals. Moreover, we have also found that the accumulation of metabolic by-products is another limiting factor of the current EVLP setting [20]. Adding a dialysis system into the EVLP circuit effectively maintained donor lung function [38]. The selected formula should be combined with the dialysis system to extend EVLP for donor lung repair, recondition, and bioengineering.

## 4. Materials and Methods

Most of the materials and methods used in the present study have been published recently [23]. We will give brief descriptions for each session.

### 4.1. Cell Culture

Human pulmonary microvascular endothelial cells (HPMEC) were a gift from Kirkpatrick’s research lab [39], and human lung epithelial cells (BEAS-2B cells) were purchased from ATCC (Manassas, VA, USA). Cells were passaged in low glucose Dulbecco’s modified Eagle’s medium (DMEM) with 10% fetal bovine serum (FBS) and Pen/Strep (100 U/100 μg/mL) (Thermo Fisher Scientific, Burlington, ON, Canada). For HPMEC cells, the flasks and plates were coated with 0.2% gelatin at least 2 h before seeding cells.

### 4.2. Automated Cellular Function Tests

The IncuCyte^®^ Live Cell Analysis System (Sartorius, Bohemia, NY, USA) was used to monitor cell confluence, apoptosis, and migration automatically. Approximately 20,000 cells/well were seeded into 96-well flat-bottomed plates, and the cell morphology was recorded in real-time up to 48 h. The IncuCyte^®^ Caspase-3/7 Green Reagent (Cat. No. 4440) was used to detect apoptosis. The IncuCyte^®^ Scratch Wound Cell Migration Assay was used to create homogenous scratch wounds in each well, and wound closure was recorded to evaluate cell migration.

### 4.3. Materials Tested to Formulate New EVLP Perfusate

Steen solution was purchased from XVIVO (Göteborg, Sweden). DMEM (low glucose Dulbecco’s modified Eagle’s medium, 11885084) was from Thermo Fisher Scientific. Dextran-40 (31389-100G) and albumin (A1653-50G) was from Sigma (St. Louise, MO, USA).

### 4.4. EVLP Cell Culture Model

The EVLP cell culture model has been described in detail [23]. Briefly, cells were cultured in 6- or 96-well plates until sub-confluent, and cold preservation was simulated by replacing serum-containing cell culture medium (DMEM plus 10% FBS) with 4 °C Perfadex^®^ preservation solution (Vitrolife, Englewood, CO, USA) with 0.3 mL/L Tham and 0.6 mL/L CaCl_2_, and cells were stored at 4 °C in a sealed chamber filled with 50% O_2_ for various time periods. Simulated EVLP was performed by replacing the cold Perfadex^®^ solution back to the Steen solution or newly designed solutions at 37 °C with 5% CO_2_ for different time periods.

### 4.5. Total Glutathione and Heat Shock Protein 70 Measurements

A Glutathione Fluorometric Assay Kit (Cat. No. K264, BioVision, Milpitas, CA, USA) was used according to manufacturer’s instructions to measure GSH. A heat shock protein 70 (HSP70) test was carried out with a kit from Abcam (Cambridge, UK), according to the manufacturer’s protocol. Please see details in a recent publication [23].

### 4.6. Statistical Analyses

Statistical analyses were performed with GraphPad prism 9.0 (GraphPad Software, San Diego, CA, USA). Two-way ANOVA was used for comparison between groups over time. The unpaired *t*-test was used for comparison between two groups. Values are presented as mean ± SD unless stated otherwise, and *p* values < 0.05 are considered statistically significant.

## 5. Conclusions

Our newly designed perfusate based on DMEM, albumin, and Dextran-40 showed promising cytoprotective effects in an EVLP cell culture model, and it should be further tested with a large animal EVLP model.

## Figures and Tables

**Table 1:** Compositions of the lung preservation solutions.

Ions (mM)	Steen	DMEM	Perfadex
Na^+^	102.2	156.2	138
K^+^	4.6	5.3	6
Ca^2+^	1.5	1.8	
Mg^2+^	1.2	0.8	0.8
Cl^−^	96	119.2	142
HCO_3_^−^	15	44	
H_2_PO_4_^−^/HPO_4_^2−^	1.2	0.9	0.8
SO_4_^2−^		0.8	0.8
Fe^3+^		2.5	
NO_3_^−^		7.5	
Glucose (mM)	11	5.5	5.5
Dextran-40	5 g/L		50 g/L
Albumin	70 g/L		
Amino acids		*	
Vitamins		*	
Others		*	
* Amino Acids (mM)		* Vitamins (mM)	
Glycine	0.4	Choline chloride	0.028571
L-Arginine hydrochloride	0.398104	D-Calcium pantothenate	0.008386
L-Cystine 2HCl	0.201278	Folic Acid	0.00907
L-Glutamine	4	Niacinamide	0.032787
L-Histidine hydrochloride-H2O	0.2	Pyridoxine hydrochloride	0.019608
L-Isoleucine	0.801527	Riboflavin	0.001064
L-Leucine	0.801527	Thiamine hydrochloride	0.011869
L-Lysine hydrochloride	0.797814	i-Inositol	0.04
L-Methionine	0.201342		
L-Phenylalanine	0.4	* Others (mM)	
L-Serine	0.4	Phenol Red	0.039851
L-Threonine	0.798319	Pyruvate	1
L-Tryptophan	0.078431		
L-Tyrosine disodium salt dihydrate	0.398467		
L-Valine	0.803419		

* the components only exist in DMEM.

## Data Availability

All data obtained in this study are provided in this manuscript.
